# Does psychological stress in patients with clinically suspect arthralgia associate with subclinical inflammation and progression to inflammatory arthritis?

**DOI:** 10.1186/s13075-018-1587-y

**Published:** 2018-05-03

**Authors:** Aleid C. Boer, Robin M. ten Brinck, Andrea W. M. Evers, Annette H. M. van der Helm-van Mil

**Affiliations:** 1 Department of Rheumatology, Leiden University Medical Centre, P.O. Box 9600, 2300 RC Leiden, The Netherlands; 2 Department of Health, Medical and Neuropsychology, Faculty of Social and Behavioural Science, Leiden University and Department of Psychiatry, Leiden University Medical Centre, Leiden, The Netherlands; 3 Department of Rheumatology, Erasmus Medical Centre, Rotterdam, The Netherlands

**Keywords:** Inflammation, Rheumatoid arthritis, C-reactive protein (CRP), Magnetic resonance imaging (MRI), Psychological status

## Abstract

**Background:**

Within established rheumatoid arthritis (RA), stress can have pro-inflammatory effects by activating the immune system via the hypothalamic-pituitary-adrenal axis and the autonomic nervous system. It is unknown if stress levels also promote inflammation during RA development. We studied whether the psychological stress response was increased in clinically suspect arthralgia (CSA) and if this associated with inflammation at presentation with arthralgia and with progression to clinical arthritis.

**Methods:**

In 241 CSA patients, psychological stress was measured by the Mental Health Inventory (MHI-5) and the Perceived Stress Scale (PSS-10) at first presentation and during follow-up. Systemic inflammation was measured by C-reactive protein (CRP) and joint inflammation by 1.5 T-MRI of wrist, MCP, and MTP joints.

**Results:**

At baseline, 12% (24/197) of CSA patients had a high psychological stress response according to the MHI-5. This was not different for patients presenting with or without an elevated CRP, with or without subclinical MRI-detected inflammation and for patients who did or did not develop arthritis. Similar findings were obtained with the PSS-10. When developing clinical arthritis, the percentage of patients with ‘high psychological stress’ increased to 31% (*p* = 0.025); during the first year of treatment this decreased to 8% (*p* = 0.020). ‘High psychological stress’ in non-progressors remained infrequent over time (range 7–13%). Stress was associated with fatigue (*p* = 0.003) and wellbeing (*p* < 0.001).

**Conclusions:**

Psychological stress was not increased in the phase of arthralgia, raised at the time of diagnoses and decreased thereafter. The lack of an association with inflammation in arthralgia and this temporal relationship, argue against psychological stress having a significant contribution to progression from CSA to inflammatory arthritis.

**Electronic supplementary material:**

The online version of this article (10.1186/s13075-018-1587-y) contains supplementary material, which is available to authorized users.

## Background

In chronic inflammatory diseases like rheumatoid arthritis (RA), psychological stress is considered to negatively affect the disease course. It activates the hypothalamic-pituitary-adrenal axis and the autonomic nervous system, which associate with the release of neurotransmitters (i.e. norepinephrine), hormones (i.e. cortisol) and activation of immune cells [1–3]. During stress, the normal downregulation of the inflammatory response is hindered, possibly causing pro-inflammatory effects. Despite these effects, it is presently unknown if stress mediates the development of early inflammatory arthritis or RA.

The prevalence and the effects of psychological stress have been thoroughly investigated within patients with established RA. Compared to the general population, RA patients experience more stress [4]. Moreover the stress response is deranged, especially in patients with increased disease activity scores and disease exacerbations [5–8]. Patients have increased stress-induced inflammatory cytokine levels (i.e., interleukin (IL)-6, IL-1b, IL-2) and impairments in the capacity of glucocorticoids to inhibit this inflammatory response [9–11]. Likewise, psychological stress has been associated with increased C-reactive protein (CRP) levels [1, 12]. Furthermore, research in patients with established RA has indicated that pre-existing inflammation can facilitate stress-induced inflammation [2, 12], potentially inducing a vicious circle between psychological stress and inflammation. Notably, the pro-inflammatory effects observed in these studies were independent of the disease duration [6, 13, 14].

The developmental course of RA is incompletely understood, though it is recognized that it consists of several phases [15]. The phase preceding that of clinically apparent (chronic) arthritis is a symptomatic one, called clinically suspect arthralgia (CSA). CSA patients have a combination of clinical characteristics that are recognizable by rheumatologists, and are at risk to develop RA [16]. These characteristics were also recently described by a EULAR taskforce [17]. Part of the CSA patients have subclinical joint inflammation that is detectable by MRI at first presentation to a rheumatologist [18], and subclinical inflammation is present in the vast majority of those CSA patients that progress to RA [16].

It is presumed that biologic mechanisms evolving in this symptomatic pre-arthritis phase of RA are important for the future course of the disease. Whether psychological stress is associated with inflammation in the phase of CSA or with progression to clinically apparent arthritis or RA is presently unknown and subject of this study. Based on the abovementioned observations in patients with established RA, stress might contribute to the development of subclinical inflammation and subsequently mediate progression to RA. The recent observation that life events pose a (small) risk at development of RA fits into this hypothesis [5]. However alternatively, stress can also be a consequence of symptoms and physical limitations without having exacerbating effects on inflammation in the phase of CSA. To increase the comprehension of the effects of the perceived psychological stress response in a symptomatic pre-arthritis phase, this study aimed to determine associations and time-relationships between stress and inflammation in patients presenting with CSA and during progression to early clinical arthritis. The psychological stress response was measured by two validated questionnaires, the five-item Mental Health Inventory (MHI-5) and Cohen’s perceived stress scale (PSS-10) [19–21]. Inflammation was measured by systemic inflammation assessed using C-reactive protein (CRP) levels and subclinical joint inflammation determined using magnetic resonance imaging (MRI) of hand and foot joints.

## Methods

### Patient population

Patients included in the Leiden Clinically Suspect Arthralgia (CSA) cohort between April 2012 and March 2015 were studied. The CSA cohort is a population-based inception cohort that started at the rheumatology outpatient clinic in Leiden, The Netherlands, with the aim of studying the symptomatic phase of RA that precedes clinical arthritis. Inclusion required the presence of arthralgia of small joints for < 1 year which was because of the character of the symptoms, considered as being suspect to progress to RA by a rheumatologist. A detailed description is provided elsewhere [16], but identification of CSA occurred mainly by the clinical expertise of the rheumatologist. Furthermore, CSA was identified at the first visit, before the results of routine laboratory investigations were known. Notably, general practitioners in our region are discouraged to perform anti-citrullinated protein antibodies (ACPA) testing themselves but are encouraged to refer in a case of any suspicion on imminent RA. After inclusion in the CSA cohort, routine visits were performed after 4 months, 1 year and 2 years. At the request of patients (e.g. in case they experienced more symptoms) patients were also seen in between the scheduled visits. All patients were followed for development of clinically apparent arthritis for 2 years. Follow-up in the CSA cohort ended earlier when clinical synovitis had developed, confirmed with joint swelling at physical examination by the treating rheumatologist. CSA patients were not treated with disease-modifying anti-rheumatic drugs (DMARDs) or corticosteroids in the phase of CSA. For the patients that developed clinical synovitis, further data was obtained as they were subsequently included in the Leiden Early Arthritis Clinic (EAC) cohort [22]. Data collected at clinical arthritis onset and 1 year thereafter were used. Patients who were diagnosed with RA were treated in conformance with national guidelines, which consists of early initiation with a DMARD (preferably methotrexate), in case of failure a second conventional DMARD (either switching or adding) and disease activity score (DAS)-steered treatment adjustments. Biologics were allowed if ≥2 conventional DMARDs failed but that this did not occur in the studied period of 1 year after clinical arthritis onset. A flowchart of the study protocol is available in Additional file 1: Figure S1. Written informed consent was obtained from all patients. The study was approved by the local medical ethics committee.

### Study protocol

At each visit physical examinations, including 66-swollen and 68-tender joint counts (66-SJC and 68-TJC) were performed and blood samples were taken to measure CRP (positive if ≥5 mg/L); immunoglobulin M-rheumatoid factor (RF) (positive if ≥3.5 IU/mL); and ACPA (anti-CCP2, EliA CCP, Phadia, The Netherlands, positive if ≥7 U/mL). Questionnaires were completed, including the health assessment questionnaire (HAQ)-disability index (DI), 36-item Short Form Health Survey (SF-36) and self-reported wellbeing, pain and fatigue on numerical rating scales ranging from 0 (no complaints) to 10 (extreme complaints). The PSS-10 was added later to the protocol in 2013 and was gathered only at baseline. Further, to measure subclinical joint inflammation, MRI scans of metacarpophalangeal (MCP), wrist and metatarsophalangeal (MTP joints were made of the most affected side (or the dominant side in case of equally severe symptoms) on an MSK Extreme 1.5 T extremity MR-system (GE, \Milwaukee, WI, USA). MRI scans were made of the same side at baseline and at arthritis onset (before the start of disease-modifying drugs including corticosteroids) and 1 year thereafter. Non-steroidal anti-inflammatory drugs (NSAIDs) were stopped 24 h before the MRI scan. The scans were scored for MRI-detected inflammation according to the Outcome Measures in Rheumatology Clinical Trials (OMERACT) RA MRI scoring (RAMRIS) method as described supplementary and previously published [16]. Total inflammation scores consisted of the sum of synovitis, bone marrow edema (BMO) and tenosynovitis scores. All scans were scored by two independent readers and mean scores of both readers were calculated to obtain the total inflammation score (see Additional file 1: Supplementary Methods). The cut-off of MRI positivity was based on healthy controls as described previously [23]. An MRI was considered positive for subclinical inflammation (tenosynovitis, synovitis or BMO) if this was present in < 5% of healthy volunteers (an example is provided in the Additional file 1: Supplementary Methods).

### Psychological stress questionnaires

The psychological stress response was measured by two questionnaires. First, for the main analyses we used the MHI-5 which is a component of the SF-36 questionnaire [19, 24]. Baseline MHI-5 was missing in 18% of the CSA patient and no differences were found in baseline characteristics between patients with an available and missing MHI-5 (Additional file 1: Table S1). The MHI-5 is a brief self-administered questionnaire which includes scales to screen for anxiety and depression [20, 25]. It is well-validated with good psychometric properties for detecting Diagnostic and Statistical Manual of Mental Disorders (DSM-IV) type I axis diagnoses [26], these disorders include clinical mental disorders like anxiety and depression [27]. The MHI-5 has a Cronbach’s α between 0.74 and 0.90 [19, 26]. The items were scored on a six-point frequency rating scale and questions are provided in the Additional file 1: Supplementary Methods. After linear conversion, possible scores on the MHI-5 range from 0 to 100, with lower scores reflecting a higher stress response. As the score has been considered as a dichotomous variable (absence or presence of a psychological stress response), a score ≥ 52 indicated minimal psychological stress (anxiety or depression) and < 52 high psychological stress; here simply called ‘high stress’. The application of this questionnaire to screen for stress by means of depression and anxiety has been examined thoroughly, as illustrated by several studies [25, 26, 28].

Second, to investigate the main results on stress further we analysed the PSS-10. As this questionnaire was added to the protocol at a later time point, PSS-10 data was missing in 44% of CSA patients. No differences were found in baseline characteristics between patients with an available and missing PSS-10 (Additional file 1: Table S2). Patients filled in a Dutch translation which consisted of ten items regarding predictability, controllability and life overload as perceived by the individual during the last month [21]. Questions are provided in the Additional file 1: Supplementary Methods and each item of the questionnaire was rated on a five-point Likert-type scale (0 = never, 1 = almost never, 2 = sometimes, 3 = fairly often, 4 = very often) how they felt with a maximum score of 40. Total scores were calculated after reversing positive items’ scores (questions 4, 5, 7, 8) and then summing up these scores with the negative items’ scores [21]. A higher total score indicates a greater perceived stress response. As there is no predetermined cut-off of high psychological stress, results on this questionnaire were not dichotomized. As reference, the mean score of PSS-10 obtained in 2387 respondents in the United States was 12.1 for males and 13.7 for females [29].

### Statistics

We tested dichotomous outcomes of markers of inflammation (CRP positivity, MRI positivity and arthritis onset). Associations at baseline were tested with logistic regression with MHI-5 as dependent variable and with linear regression with PSS-10 as dependent variable. Longitudinal data of the MHI-5 were assessed with logistic regression with a generalized estimating equation (GEE) model with an unstructured matrix and a logit link function. Longitudinal data on CRP levels and MRI-detected inflammation scores were analysed with a GEE model with an unstructured matrix. In all analyses, (at baseline and follow-up) we corrected for age and gender. Further, as sub-analysis, we repeated analyses in CSA patients that also fulfilled the EULAR definition of arthralgia suspicious for progression to RA. To fulfil the definition patients should have ≥ 3 of these characteristics: joint symptoms of recent onset (duration < 1 year), symptoms of MCP joints, morning stiffness ≥60 min, most severe symptoms in early morning, presence of a first-degree relative with RA, difficulty with making a fist and a positive squeeze test of MCP joints [17]. IBM SPSS v23 (IBM Corp, Armonk, NY, USA) was used. The significance level was set at 0.05. To adjust for multiple testing for the ten different comparisons made at baseline, we applied Bonferroni correction and then *p* values < 0.005 were considered significant.

## Results

### Patient characteristics

Table 1 presents the baseline characteristics of the 241 CSA patients. The mean age was 44 and the majority was female. Forty-five patients developed clinical synovitis after a median follow-up of 17 weeks; 65% of these patients fulfilled the 2010 criteria and 91% started DMARD therapy.

**Table 1:** Baseline characteristics of all 241 CSA patients studied and of the 186 patients that also completed the PSS-10

	MHI-5 (*n* = 241)	PSS-10 (*n* = 186)
Age, mean (SD)	44 (9)	44 (13)
Female, n (%)	187 (78)	147 (79)
68-tender joint count, median (IQR)	6 (3–11)	5 (3–10)
CRP (mg/L), median (IQR)	3 (3–5)	3 (3–5)
RF positive (≥ 3.5 IU/mL), n (%)	51 (21)	37 (20)
ACPA positive (≥ 7 U/mL), n (%)	32 (13)	21 (11)
Symptom duration in weeks, median (IQR)	17 (9–31)	18 (10–31)

*ACPA* anti-citrullinated peptide antibody, *CRP* C-reactive protein, *IQR* interquartile range, *RF* rheumatoid factor, *SD* standard deviation

### Stress measured at presentation with CSA

**Fig. 1:**
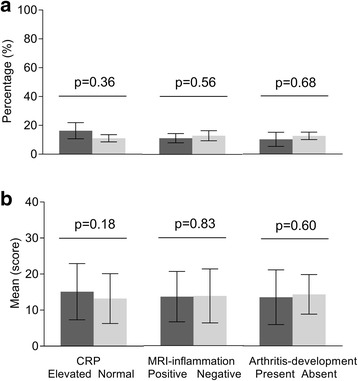
Percentages of patients with high psychological stress measured by MHI-5 (**a**) and obtained mean PSS-10 scores (**b**) in patients who at presentation with CSA had elevated versus normal CRP levels, did or did not have MRI-detected subclinical joint inflammation and patients who did and did not progress to clinical arthritis over time. In *A* the percentage of patients with high perceived psychological stress by MHI-5 (score < 52) are shown. These were not significantly different between patients with elevated versus normal CRP (7/43 versus 17/154), with or without MRI-detected inflammation (11/97 versus 12/92) and between patients who did and did not progress to clinical arthritis over time (4/39 versus 20/158)). In *B* the values of psychological stress by PSS-10 are shown. These results were similar. In *A* whiskers indicate standard error and in *B* whiskers indicate standard deviation

At presentation with CSA, 10% (4/39) of patients with arthritis during follow-up and 12% (24/197) of the total group of CSA patients had a high psychological stress response (‘high stress’) according to the MHI-5 (score < 52). This was not different for patients presenting with or without an elevated CRP (16 versus 11%, *p* = 0.36), or with or without subclinical MRI-detected inflammation (11 versus 13%, *p* = 0.56, Fig. 1). Also for the continuous values of CRP and MRI-detected inflammation scores with stress we did not find any significant relationships (*p* = 0.89 and *p* = 0.90, respectively). Further, we did not find associations between stress and age, gender, 68-TJC, self-reported pain, or HAQ-DI. Significant associations were observed between stress and self-reported fatigue (odds ratio [OR] = 1.5, (95% confidence interval (95% CI) 1.1; 1.9); *p* = 0.003) and wellbeing (OR = 1.6, (95% CI 1.3; 2.1); *p* < 0.001). This means that patients with a high stress response had 1.5 times more severe fatigue and they felt 1.6 times more severely affected in their general wellbeing compared to patients without ‘high stress’.

When analysing the continuous values of the MHI-5, instead of dichotomized, this showed similar results. We found no association for patients presenting with or without an elevated CRP (*p* = 0.15), or with or without subclinical MRI-detected inflammation (*p* = 0.60) and also not between stress and age, gender, 68-TJC and self-reported pain. The association between self-reported fatigue (β = − 2.5 (95% CI -3.4; − 1.5); *p* < 0.001) and wellbeing (β = − 3.4 (95% CI -4.5; − 2.3); *p* < 0.001) were also found here and additionally we identified associations between stress and HAQ-DI (β = − 9.2 (95% CI −14.6; − 3.8); *p* = 0.001). The latter means that per point increase in functional disability patients experienced more severe stress, reflected by a 9.2 lower MHI-5 score (on a range 0–100).

When analysing the outcome arthritis-onset during follow-up, there was no association between the percentage of patients with a high stress response at baseline and the number of patients who did and did not develop clinical arthritis (10 versus 13%, *p* = 0.68, Fig. 1). Also for the continuous MHI-5 score this relationship was non-significant (*p* = 0.71).

### Stress measured during progression to clinical arthritis

**Fig. 2:**
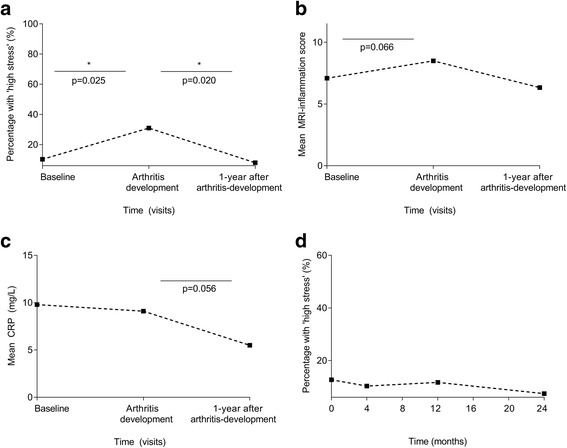
Longitudinal data of percentages of patients with high psychological stress by MHI-5 (**a**), total MRI inflammation scores (**b**) and CRP levels (**c**), in patients presenting with CSA, and during and after the development of clinical synovitis (**a**, **b**, **c**) and frequency of patients with ‘high stress’ over time in CSA patients that did not progress to clinical synovitis (**d**). In *A* the percentages of patients with high stress, with all patients who progressed to clinical arthritis as reference, at baseline 10% (4/39) had ‘high stress’, at arthritis onset 31% (9/29) and 1 year thereafter 8% (2/25). In *B* the MRI-detected inflammation scores increased during progression to clinical arthritis (*p* = 0.066); the number of MRI scans at the final time point is limited, hence these data were not incorporated in statistical analyses. In *D* the percentages of patients with high stress, among all CSA patients who did not develop clinical arthritis during 2 years of follow-up (*n* = 196) are shown; this percentage remained rather stable (*p* = 0.42)

At the time of arthritis-onset, the percentage of patients with a high psychological stress response increased to 31% (*p* = 0.025). One year later this had decreased to 8% (*p* = 0.020, Fig. 2).

Also, inflammation was measured over time in patients that progressed to clinical arthritis. The CRP levels were stable while progressing from CSA to clinical arthritis (mean 9.8 and 9.1 mg/L respectively, *p* = 0.83) and decreased during the first year of treatment (mean 5.5 mg/L; *p* = 0.056). Total MRI-detected joint inflammation -scores increased between presentation with CSA and arthritis-onset, though this did not reach statistical significance (mean score 7.1 and 8.5 respectively, *p* = 0.066). One year after presentation with clinical arthritis the scores had decreased (mean score 6.3).

Then we investigated whether baseline stress associated with the MRI-detected total inflammation score or CRP at arthritis onset and this revealed non-significant relationships (*p* = 0.11 and *p* = 0.92).

### Stress measured in patients that did not progress to clinical arthritis

The percentage of patients with a high psychological stress response among the CSA patients that did not progress to clinical arthritis during 2-years of follow-up (*n* = 196) was stable and ranged between 7 and 13% (*p* = 0.42, Fig. 2).

### Sub-analyses

To verify the main findings done on psychological stress present at presentation with CSA, we also analysed stress responses measured by the PSS-10. The mean PSS-10 score in all patients was 13.5 (SD 7.6). Patients that presented with or without an elevated CRP, with or without subclinical MRI-detected inflammation or who did and did not develop arthritis did not have higher stress levels (*p* = 0.18, *p* = 0.83 and *p* = 0.60 respectively, Fig. 1). We observed significant positive associations at baseline between stress and self-reported fatigue (β = 0.94; *p* < 0.001), wellbeing (β = 1.20; *p* < 0.001).

Additionally, analyses were repeated in patients that were identified as CSA by their rheumatologist and also fulfilled the EULAR definition for CSA (cut-off ≥ 3 items present), which was fulfilled by 75% of the CSA patients. The main findings were similar here too. Measuring the stress response by MHI-5 revealed that the percentage of patients with ‘high stress’ was not statistically different between patients presenting with or without an elevated CRP (16 versus 12%, *p* = 0.53), with or without MRI-detected inflammation (14 versus 12%, *p* = 0.92) or for patients who did and did not develop arthritis (13 versus 13%, *p* = 0.92). Also when measuring stress levels with the PSS-10 no differences were observed for patients with elevated versus normal CRP, positive or negative MRI-detected inflammation and between patients with and without clinical arthritis during follow-up (*p* = 0.34, *p* = 0.91 and *p* = 0.61, respectively, Additional file 1: Figure S2). Also in this subgroup of patients, association between stress (according to both questionnaires) and wellbeing was statistically significant (*p* < 0.001). Repeating the longitudinal analyses on stress in the subgroup of patients that fulfilled the EULAR definition revealed similar findings as in the total group; both for the patients that progressed to clinical arthritis as in those that did not progress (Additional file 1: Figure S3).

## Discussion

This longitudinal study assessed associations and time-relations between the psychological stress response and inflammation in an early symptomatic phase of development of RA. We found no relationship between stress and either local or systemic inflammation in patients with clinically suspect arthralgia and also no association between stress and future clinical arthritis development. However, levels of psychological stress were increased at the visit when clinical arthritis was identified and decreased during the first year of treatment. This is the first study that evaluated stress in a symptomatic pre-arthritis phase. Although association studies cannot prove causality, the present data on the time-relationship suggest that stress may be more a consequence of symptoms and physical limitations related to the occurrence of early clinical arthritis, or of concerns related to the diagnosis that has just been made by a rheumatologist, rather than a cause for the development of inflammatory arthritis or RA.

At presentation with CSA 10% of the patients with arthritis during follow-up had high stress-levels; this percentage is similar to that of the general population [24, 30, 31]. Also, the mean PSS-10 levels that we observed (13.5) were in line with those previously reported in the USA [21, 29].

Previously, we observed that patients with CSA had increased pain levels and that levels of functional disability already equalled those at the phase of clinically apparent arthritis. Hence, even though patients with CSA do experience significant pain and physical limitations [32], stress levels were not evidently increased. Thus, although studies in established RA revealed associations between pain and stress, in the pre-RA stage of CSA the symptoms themselves apparently did not result in higher stress levels.

Our longitudinal analyses revealed that at the time of presentation with clinical arthritis, which generally is the time when a diagnosis is established, 31% of the patients experienced a high stress response. This prevalence is similar to that observed in studies on (established) RA [24, 31, 33].

Multiple studies performed in established RA showed associations between stress (both short-lived stress induced in an experimental setting and stress experienced in real life) and inflammation [1, 2, 6, 9–14]. Clear associations were observed for different markers of systemic inflammation (CRP and pro-inflammatory cytokines). Associations with DAS were also observed; interestingly these associations were stronger with the subjective parameters of the DAS, specifically patient’s global assessment, evaluator’s global assessment and TJC, whereas they found no clear associations with SJC and acute phase reactants [34]. MRI-detected joint inflammation has never been studied before in relation to the psychological stress response, neither in patients with CSA nor in patients with clinically apparent arthritis or RA. Thus the current observations on inflammation and stress in CSA are different from that previously reported in patients with established RA.

The stress response was assessed using two questionnaires. The main analyses were performed using data obtained by the MHI-5 and results were verified by the PSS-10. Both questionnaires are brief and have been shown to have good concordance with larger questionnaires [24]. In our study, results were similar for both questionnaires, which shows validity of the results. Furthermore, by using both questionnaires we observed associations between the psychological stress response and fatigue and general wellbeing. These associations have previously been observed in patients with RA. Thus, although the main results of this study were negative, other known associations were also present in patients with CSA.

Patients with CSA were identified by their rheumatologists using their clinical expertise. Recently, a EULAR definition for arthralgia suspicious for progression to RA was developed to be used on top of the clinical suspicion of imminent RA. This serves to reduce heterogeneity in patient groups, which is highly relevant for the execution of scientific studies and clinical trials in particular [17, 35]. In this study, we repeated the analyses in CSA patients that fulfilled this EULAR definition and similar results were obtained.

Not only stress, but also inflammation was measured over time. Joint inflammation was evaluated using MRI. As expected, the MRI inflammation score increased during progression to clinical arthritis and decreased during the first year of treatment. Despite a strong tendency in the data, statistical significance was not obtained. This is partly explained by a relatively small number of progressors. In addition, MR imaging was made unilaterally of hand and foot joints and consequently other joints that developed clinical arthritis were not imaged. Third, the serial MRIs were scored without information on time order; this decreased the sensitivity to detect changes over time and may have resulted in lower scores compared to chronological reading [36]. Importantly, serial MRIs were not primary evaluated to determine statistical significant changes in the course of MRI-detected inflammation, but rather to compare the time course of stress to that of the course of inflammation.

This study had some limitations. As for the presence of missing data, 82% of patients completed the MHI-5 and patients with missing data did not differ from patients who completed this questionnaire. Data on the PSS-10 was missing in the oldest part of the cohort, hence missingness was completely at random. Second, the role of acute and chronic stress on inflammation may be complex [3], here we measured the psychological stress experienced by patients during the last month. We did not collect data on other psychological factors (e.g. coping mechanisms, social support, psychiatric comorbidities) or life events to account for in measurements that could have played a role in arthritis commencement. Also the occurrence of major life events in childhood or more recently in adulthood was not specifically investigated and effects of such major stressors were not evaluated in this study. In addition, regarding systemic inflammation, we only determined CRP levels and did not evaluate other markers, like cytokines. In addition, cortisol or other hormones were not assessed. Further, an important remark could be that CRP values can be increased by diseases not related to the joints. Only 27 of 241 patients had CRP values > 10; one of these patients had a comorbidity that may have influenced the CRP level (benign prostate hypertrophy with chronic inflammation). Exclusion of this patient did not change the results (data not shown). Finally, early identification of CSA is difficult, and therefore the size of the current study (241 CSA patients with longitudinal follow-up) is considerable. However, power to detect associations with stress with very small effect sizes may have been insufficient. The previously observed association between life events and risk for RA reported an OR of 1.1 and effects of this size will remain undetected in the present data.

## Conclusions

In conclusion, this study is the first that evaluated the stress response in a symptomatic pre-arthritis phase and also contained longitudinal data. In the phase of arthralgia, high stress levels were infrequent. The proportion of patients with a high stress response increased at the time of clinical arthritis development and diagnosis. Hence, the course of stress levels paralleled or followed, but not preceded, the course of inflammation in this study. This temporal relationship as well as the lack of an association of stress with local or systemic inflammation in the phase of arthralgia may suggest that in this very early disease phase when disease chronicity has not yet been established, stress may have little influence on the inflammatory response, and therefore this implies that it does not mediate the progression from arthralgia to clinical arthritis. Although further studies on the association of stress and inflammation in pre-RA are required, the vicious circle of stress and inflammation as observed in patients with established RA was not yet observed in the phase of CSA.

## Additional file

Additional file 1: Supplementary material. (DOCX 195 kb)

## Abbreviations

1.5 T: 1.5 Tesla
ACPA: Anti-citrullinated protein antibodies
BMO: Bone marrow edema
CRP: C-reactive protein
CSA: Clinically suspect arthralgia
DAS: Disease activity score
DMARD: Disease-modifying antirheumatic drug
GEE: Generalized estimating equation
HAQ-DI: Health assessment questionnaire-disability index
IL: Interleukin
IQR: Interquartile range
MCP: Metacarpophalangeal
MHI-5: Mental health inventory
MRI: Magnetic resonance imaging
MTP: Metatarsophalangeal
OMERACT: Outcome Measures in Rheumatology Clinical Trials
OR: Odds ratio
PSS-10: Cohen’s perceived stress scale
RA: Rheumatoid arthritis
RAMRIS method: RA MRI scoring method
RF: Rheumatoid factor
SD: Standard deviation
SF-36: 36-item Short Form Health Survey
SJC: Swollen joint count
TJC: Tender joint count
