# Supplementary material for: Does psychological stress in patients with clinically suspect arthralgia associate with subclinical inflammation and progression to inflammatory arthritis?
Source: Arthritis Res Ther. 2018 May 3;20:93. doi: 10.1186/s13075-018-1587-y (PMC5934795; doi:10.1186/s13075-018-1587-y)
Supplement: Supplementary file 1 — Supplementary material. (DOCX 195 kb) [file 13075_2018_1587_MOESM1_ESM.docx]

**Additional file 1**

**Figure S1.** Study protocol.

MRI

Laboratory investigations

MHI-5 Questionnaire

PSS-10 Questionnaire

Laboratory investigations

MHI-5 Questionnaire

MRI

Laboratory investigations

MHI-5 Questionnaire

Laboratory investigations

MHI-5 Questionnaire

Baseline visit in Clinically Suspect Arthralgia cohort

Follow-up visit in Early Arthritis Clinic cohort*

Baseline visit in Early Arthritis Clinic cohort

Follow-up visits in Clinically Suspect Arthralgia cohort†

*Arthritis*

*Development*

Legend. Inclusion in Clinically Suspect Arthralgia (CSA) cohort at first presentation to outpatient clinic after onset of arthralgia complaints (n=241). Follow-up visits in CSA-cohort at 4 months, 1 year and 2 years. Patients that developed arthritis (n=45) during follow-up were included in the Early Arthritis Clinic (EAC) cohort (n=39).

† In case of no arthritis development follow-up ends after 2 years; *Only 1 year visit was studied here.

**Figure S2.** Percentages of patients with high psychological stress measured by MHI-5 (A) and obtained mean PSS-10 scores (B) in patients that at presentation with CSA that fulfilled the EULAR-criteria for arthritis suspicious for progression to RA that had elevated versus normal CRP-levels, did or did not have MRI-detected subclinical joint inflammation and patients that did and did not progress to clinical arthritis over time.


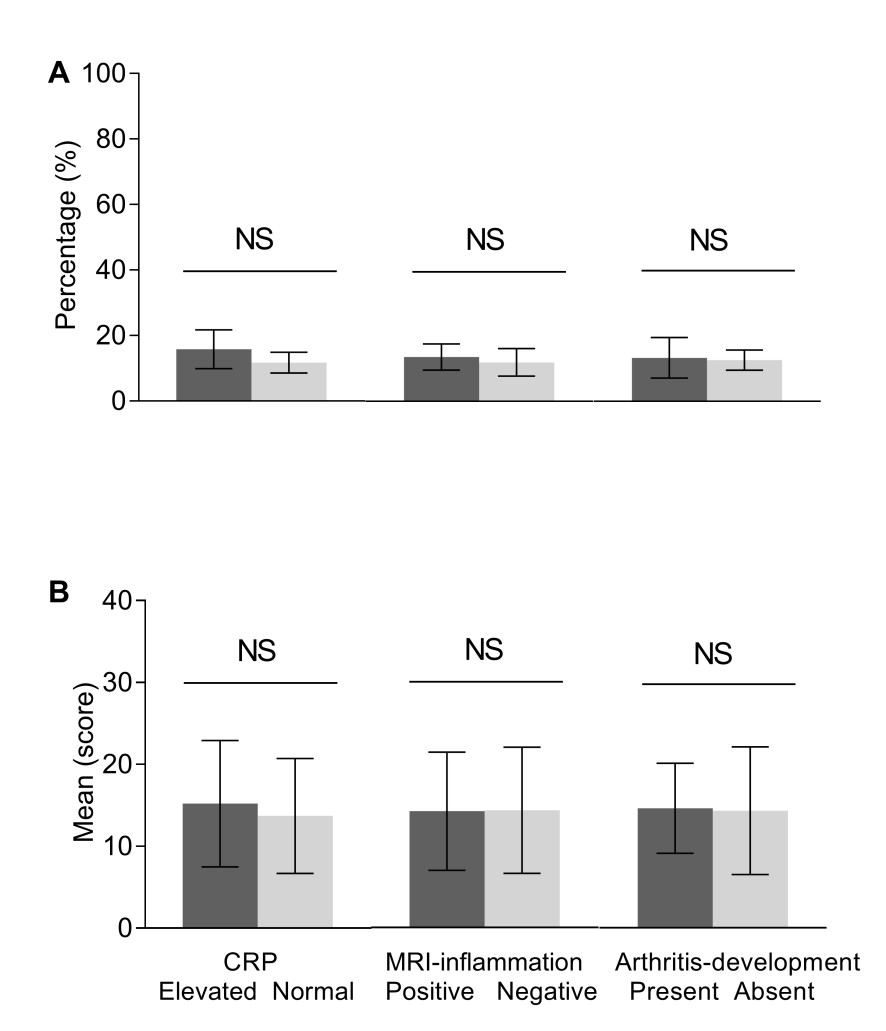


In A whiskers indicate standard error and in B whiskers indicate standard deviation. NS indicates non-significance.


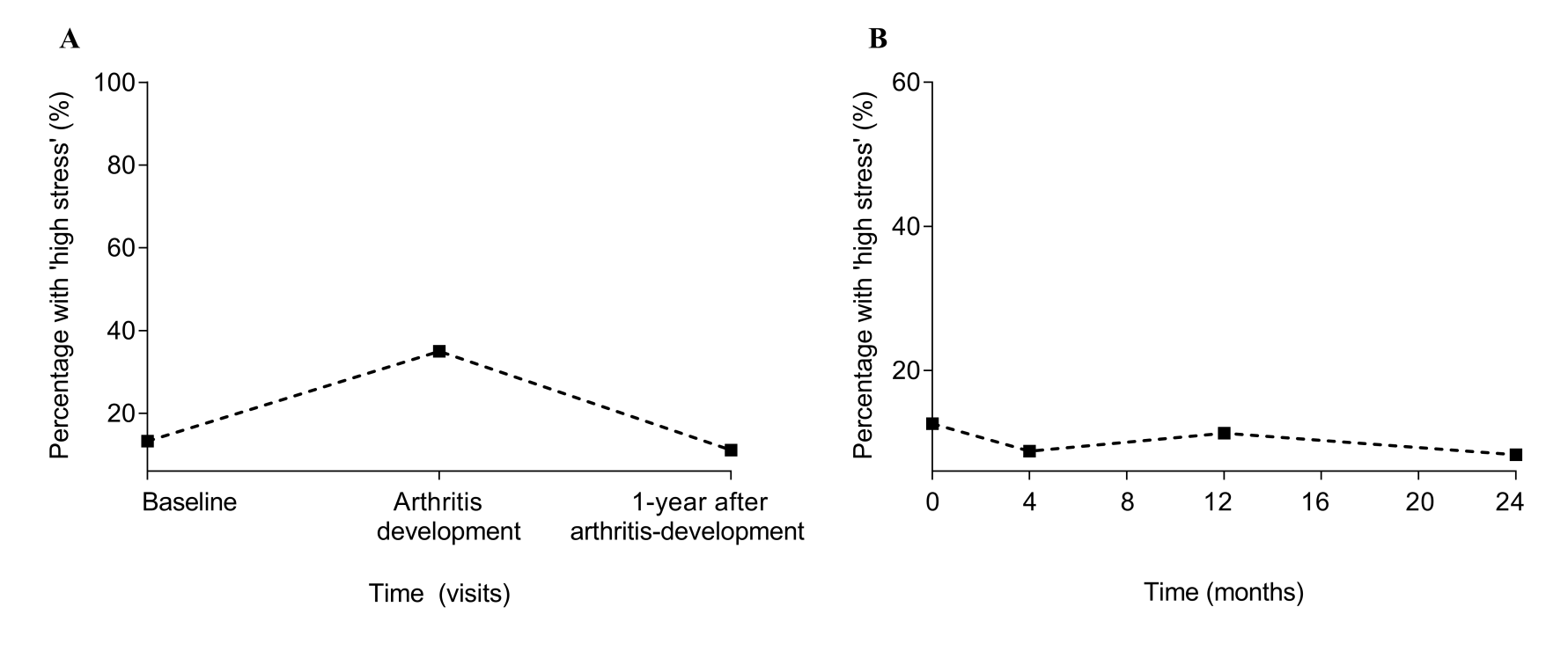
**Figure S3.** Percentages of CSA patients that fulfilled the EULAR-definition and that perceived high psychological stress at presentation with CSA an during progression to clinical arthritis (A) or during follow-up without arthritis development (B)

Table S1. Characteristics of patients with available and missing baseline data of MHI-5.

|  | **Missing-data**  **(n=44)** | **Available-data**  **(n=197)** | **P-value** |
| --- | --- | --- | --- |
| Age, mean (SD) | 44 (12) | 44 (13) | 0.81 |
| Female, n (%) | 36 (82) | 151 (77) | 0.46 |
| 68-Tender joint count, median (IQR) | 7 (3-15) | 5 (2-10) | 0.14 |
| CRP (mg/L), median (IQR) | 3 (3-5) | 3 (3-5) | 0.64 |
| RF-positive, n (%) | 11 (25) | 40 (20) | 0.49 |
| ACPA-positive, n(%) | 9 (21) | 23 (12) | 0.12 |
| Symptom duration in days, median (IQR) | 102 (20-113) | 108 (52-114) | 0.13 |

P-values calculated with Mann-Whitney U test, dichotomous outcomes with Chi-square test and age with independent t-test. Differences were not statistically significant.

SD, standard deviation; IQR, Inter quartile range; CRP, c-reactive protein; RF, rheumatoid factor; ACPA, anti-citrullinated protein antibody.

Table S2. Characteristics of patients with available and missing baseline perceived stress scale-10 data.

|  | **Missing-data**  **(n=83)** | **Available-data**  **(n=103)** | **P-value** |
| --- | --- | --- | --- |
| Age, mean (SD) | 45 (12) | 44 (13) | 0.65 |
| Female, n (%) | 68 (82) | 79 (77) | 0.38 |
| 68-Tender joint count, median (IQR) | 6 (3-10) | 5 (2-10) | 0.73 |
| CRP (mg/L), median (IQR) | 3 (3-5) | 3 (3-5) | 0.68 |
| RF-positive, n (%) | 17 (21) | 20 (19) | 0.86 |
| ACPA-positive, n(%) | 9 (11) | 12 (12) | 0.86 |
| Symptom duration in days, median (IQR) | 107 (22-113) | 106 (41-114) | 0.72 |

P-values calculated with Mann-Whitney U test, dichotomous outcomes with Chi-square test and age with independent t-test. Differences were not statistically significant.

SD, standard deviation; IQR, Inter quartile range; CRP, c-reactive protein; RF, rheumatoid factor; ACPA, anti-citrullinated protein antibody.

Table S3. Characteristics of patients with (<52) and without (≥52) high perceived stress by the MHI-5.

|  | **No ‘high stress’ (≥52)**  **(n=173)** | **‘High stress’ (<52) (n=24)** |
| --- | --- | --- |
| Age, mean (SD) | 44 (13) | 47 (11) |
| Female, n (%) | 131 (76) | 20 (83) |
| 68-Tender joint count, median (IQR) | 5 (2-11) | 6 (4-9) |
| CRP (mg/L), median (IQR) | 3 (3-4) | 4 (3-7) |
| RF-positive, n (%) | 32 (19) | 8 (33) |
| ACPA-positive, n(%) | 19 (11) | 4 (17) |
| Symptom duration in days, median (IQR) | 18 (9-33) | 23 (9-40) |

SD, standard deviation; IQR, Inter quartile range; CRP, c-reactive protein; RF, rheumatoid factor; ACPA, anti-citrullinated protein antibody.

**SUPPLEMENTARY METHODS**

***Stress questionnaires***

*MHI-5*

This questionnaire is a part of the SF-36 and contains a Dutch translation of the following questions: Over the last month, how often have you: (9b) been a very nervous person? (9f) felt downhearted and blue? (9d) felt calm and peaceful? (9c) felt so down in the dumps that nothing could cheer you up? (9h) been a happy person?

*PSS-10*

This questionnaire contains a Dutch translation of the following questions: In the last month, (1) how often have you been upset because of something that happened unexpectedly? (2) how often have you felt that you were unable to control the important things in your life? (3) how often have you felt nervous and ‘stressed’? (4) how often have you felt confident about your ability to handle your personal problems? (5) how often have you felt that things were going your way? (6) how often have you found that you could not cope with all the things that you had to do? (7) how often have you been able to control irritations in your life? (8) how often have you felt that you were on top of things? (9) how often have you been angered because of things that were outside of your control? (10) how often have you felt difficulties were piling up so high that you could not overcome them?

***MR scoring***

The scoring protocol was identical for all patients as described in previous studies[1-3]. Bone marrow oedema (BMO) and synovitis were scored in line with the Outcome Measures in Rheumatology Clinical Trials (OMERACT) rheumatoid arthritis MRI scoring system (RAMRIS)[4]. Although BMO was originally scored on T2fatsat, it was shown in several settings among which early arthritis and RA that scoring BMO on post-contrast T1 can be assessed equally well[5-8]. Tenosynovitis in the MCP and wrist was scored as described by Haavardsholm et al[9]. Although RAMRIS was not developed to score MTP-joints, others have previously adapted the RAMRIS to score MTP-joints as well[9]. BMO, tenosynovitis and synovitis were scored from grade 0-3. Mean scores of two readers were calculated and in case of a score of ≥1, MRI was considered positive for MRI-detected inflammation. Total MRI-inflammation scores were obtained by adding scores of all joints, for each of the different types of inflammation.

Further, MRI-positivity on subclinical inflammation was determined by a cut-off that was based on data from healthy volunteers[2], explained in detail in reference.[10] When each of two readers indicated inflammation in at least 1 joint (for synovitis, BMO or tenosynovitis) that was present in <5% of the healthy persons in the same age-category at the same location[2], this was considered a ‘positive MRI’[10]. For example, a 65-year old patient with grade 1 synovitis in MCP-4 was considered positive for MRI-detected inflammation as it was seen in only 4% of controls in this age category on this location. In a 65-year old patient with grade 1 tenosynovitis of the flexor of MCP-3 this was considered negative for MRI-detected inflammation as this was seen in 12% of controls[2].

***MR readers***

Scoring was performed by independent and trained readers, blinded to clinical data. For the CSA cohort and the reference group the within-reader intraclass correlation coefficients (ICC) for the total MRI inflammation score were 0.99 (reader 1) and 0.98 (reader 2); the between-reader ICC was 0.96. For the EAC cohort within-reader ICC for the total MRI inflammation scores, were 0.98 (reader 3) and 0.93 (reader 4) respectively and the between-reader ICC, based on all scans was 0.95. In addition, between-reader ICC were calculated for total MRI inflammation scores and were for reader 1 and 3 as well as reader 2 and 3, 0.96, based on 40 randomly chosen MRI scans. The readers were blinded to the clinical data and chronological order of the scans.

**REFERENCES**

1. van Steenbergen, H.W., J.A. van Nies, T.W. Huizinga, J.L. Bloem, M. Reijnierse, and A.H. van der Helm-van Mil, Characterising arthralgia in the preclinical phase of rheumatoid arthritis using MRI. Ann Rheum Dis, 2015. 74(6): p. 1225-32.

2. Mangnus, L., H.W. van Steenbergen, M. Reijnierse, and A.H. van der Helm-van Mil, Magnetic Resonance Imaging-Detected Features of Inflammation and Erosions in Symptom-Free Persons From the General Population. Arthritis Rheumatol, 2016.

3. W.P. Nieuwenhuis, H.W.v.S., L.M. Mangnus , E.C. Newsum , J.L. Bloem , T.W.J. Huizinga , S. le Cessie, M. Reijnierse, A.H.M. van der Helm-van Mil, Evaluation of the diagnostic accuracy of hand and foot MRI for early Rheumatoid Arthritis. Rheumatology (Oxford), 2017.

4. Ostergaard, M., J. Edmonds, F. McQueen, C. Peterfy, M. Lassere, B. Ejbjerg, et al., An introduction to the EULAR-OMERACT rheumatoid arthritis MRI reference image atlas. Ann Rheum Dis, 2005. 64 Suppl 1: p. i3-7.

5. Stomp, W., A. Krabben, D. van der Heijde, T.W. Huizinga, J.L. Bloem, A.H. van der Helm-van Mil, et al., Aiming for a shorter rheumatoid arthritis MRI protocol: can contrast-enhanced MRI replace T2 for the detection of bone marrow oedema? Eur Radiol, 2014. 24(10): p. 2614-22.

6. Schmid, M.R., J. Hodler, P. Vienne, C.A. Binkert, and M. Zanetti, Bone marrow abnormalities of foot and ankle: STIR versus T1-weighted contrast-enhanced fat-suppressed spin-echo MR imaging. Radiology, 2002. 224(2): p. 463-9.

7. Mayerhoefer, M.E., M.J. Breitenseher, J. Kramer, N. Aigner, C. Norden, and S. Hofmann, STIR vs. T1-weighted fat-suppressed gadolinium-enhanced MRI of bone marrow edema of the knee: computer-assisted quantitative comparison and influence of injected contrast media volume and acquisition parameters. J Magn Reson Imaging, 2005. 22(6): p. 788-93.

8. Tamai, M., A. Kawakami, M. Uetani, A. Fukushima, K. Arima, K. Fujikawa, et al., Magnetic resonance imaging (MRI) detection of synovitis and bone lesions of the wrists and finger joints in early-stage rheumatoid arthritis: comparison of the accuracy of plain MRI-based findings and gadolinium-diethylenetriamine pentaacetic acid-enhanced MRI-based findings. Mod Rheumatol, 2012. 22(5): p. 654-8.

9. Haavardsholm, E.A., M. Ostergaard, B.J. Ejbjerg, N.P. Kvan, and T.K. Kvien, Introduction of a novel magnetic resonance imaging tenosynovitis score for rheumatoid arthritis: reliability in a multireader longitudinal study. Ann Rheum Dis, 2007. 66(9): p. 1216-20.

10. Boer, A.C., L.E. Burgers, L. Mangnus, R.M. ten Brinck, W.P. Nieuwenhuis, H.W. van Steenbergen, et al., Using a reference when defining an abnormal MRI reduces false-positive MRI results-a longitudinal study in two cohorts at risk for rheumatoid arthritis. Rheumatology, 2017. 56(10): p. 1700-1706.
